# Tungsten-enhanced growth of *Methanosphaera stadtmanae*

**DOI:** 10.1186/1756-0500-5-238

**Published:** 2012-05-15

**Authors:** Bédis Dridi, Saber Khelaifia, Marie-Laure Fardeau, Bernard Ollivier, Michel Drancourt

**Affiliations:** 1 Unité de Recherche sur les Maladies Infectieuses et Tropicales Emergentes UMR CNRS 6236 IDR 198, IFR48, Institut Méditerranée Infection, Aix-Marseille-Université, Marseille, France; 2 Laboratoire de Microbiologie IRD, UMR D180, Microbiologie et Biotechnologie des Environnements Chauds, Aix-Marseille-Université, ESIL, Marseille, France

**Keywords:** Methanogenic *Archaea*, *Methanosphaera stadtmanae*, *Methanomassiliicoccus luminyensis*, Tungsten, Selenium

## Abstract

**Background:**

The methanogenic *Archaea Methanosphaera stadtmanae* has been detected in the human gut microbiota by both culture and culture-independent methods. Its growth reaches an exponential phase after 5 to 7-day culture in medium 322 (10% vol). Our recent successful isolation of *Methanomassiliicoccus luminyensis*, a tungstate-selenite-requiring *Archaea* sharing similar metabolism characteristics with *M. stadtmanae* prompted us **to study** the effects of tungsten and selenium on *M. stadtmanae* growth.

**Findings:**

Addition of 0.2 mg/L sodium tungstate to medium 322 yielded, 48 hours after inoculation, a growth rate equivalent to that obtained after 6 days with control culture as measured by methane monitoring and optical density measurement. Addition of 50 μg/mL sodium selenate had no effect on *M. stadtmanae* growth. Quantitative real-time PCRs targeting the *M*. *stadtmanae* 16S rRNA confirmed these data.

**Conclusions:**

These data provide new information regarding the poorly known nutritional requirements of **the** human gut **colonizing organisms***M*. *stadtmanae*. Adding sodium tungstate to basal medium may facilitate phenotypic characterization of **this organism** and **additionally aid** the isolation of new *Archaea***from** complex host microbiota.

## Findings

*Methanosphaera stadtmanae* is a spherical-shaped, non-motile archaeon initially isolated from human feces [1]. *M. stadtmanae* was the first human *Archaea* to be genome sequenced and analysis of the genome confirmed that *M. stadtmanae* belonged to *Methanobacteriales*[2]. PCR-based analyses further indicated that *M. stadtmanae*-specific sequences could be detected in stool specimen in up to 30% of individuals [3]. However, *M. stadtmanae* is a fastidious organism, with only one *M. stadtmanae* isolate reported and accordingly only one *M. stadtmanae* strain available in public collections. *M. stadtmanae* oxidizes hydrogen to reduce methanol into methane [1,2]. This metabolic trait has been already reported for *M. stadtmanae*[4], and more recently for members of the genus *Methanobacterium* (e.g. *M. veterum* and *M. lacus*; [5,6]) within the order *Methanobacteriales*. We recently isolated *Methanomassiliicoccus luminyensis*, the first cultured representative of new order of methanoarchaea [7]. This archaeon exhibits a metabolic trait similar to that of *M. stadtmanae* by using hydrogen as electron donor and methanol as electron acceptor [7]. Unexpectedly, we observed that addition of tungstate-selenite to culture medium had been a key factor for successful isolation of *M. luminyensis* and that this archaeon indeed required tungstate-selenite as an essential element for growth. We therefore tested the hypothesis that the addition of sodium tungstate or sodium selenate or both to basal culture medium would also enhance the growth of *M. stadtmanae*.

*M. stadtmanae* DSMZ 3091 ^T^ (ATCC 43021^T^) purchased from the German Collection of Microorganisms and Cell Cultures (DSMZ, Braunschweig, Germany) was grown on medium 322 (http://www.dsmz.de) incubated at 37°C in Hungate tubes (Dutscher, Issy-les-Moulineaux, France) under 2-bar pressure of a H_2_/CO_2_ (80–20) atmosphere. The inoculated medium (10% vol) was incubated at 37°C with shaking. On the exponential phase of this first culture, a second inoculation was performed by 10% vol. in the same basal medium modified or not by the addition of Na_2_O_4_W (0.2 mg/L) and/or Na_2_O_4_Se (50 μg/L) (Sigma, Saint-Quentin Fallavier, France). Non-inoculated media were used as negative controls and each experiment was repeated ten times.

Growth was assessed by optical microscope observation, parallel methane production measurement and measurement of the optical density of the medium. Methane production measurement used a GC-8A gas chromatograph (Shimadzu, Champs-sur-Marne, France) equipped with a thermal conductivity detector and a Chromosorb WAW 80/100 mesh SP100 column (Alltech, Carquefou, France). N_2_ at a pressure of 100 kPa was used as the carrier gas. The detector and the injector temperatures were 200°C, and the column temperature was 150°C. H_2_ consumption and CH_4_ production were measured every 6 hours for 24 hours and then every 12 hours for 6 days. The optical density at 580 nm was measured by inserting Hungate tubes into the spectrophotometer (Varian Cary50; Agilent Technologies, Massy, France). Experiment was done in triplicate and average optical density value for the three replicates was calculated.

*M. stadtmanae* DNA extraction, quantification and sequencing were performed as previously described based on specific quantitative real-time PCR targeting 16S rRNA gene [3].

Negative controls (with and without tungstate and selenium) remained negative with no growth occurring after one-week incubation indicating that results herein reported did not merely result from carry-over of organisms. The exponential phase of *M. stadtmanae* growth cultured in medium 322 was reached at 6-day incubation. At this point microscopic observation disclosed organisms with morphology compatible with *M. stadtmanae* and no contaminant. Also, qPCR detected an equivalent of 3.22E + 12 ± 1.53E + 11 copies of 16S rRNA gene/mL (Table 1). Sequencing of 16S rRNA gene PCR products from all specimens yielded a sequence similarity of 99-100% with the reference *M. stadtmanae* DSM 3091 sequence.

**Table 1:** ** *M. stadtmanae* 16S rDNA gene copy number after 48-hour culture with Na**_**2**_**O**_**4**_**W + Na**_**2**_**O**_**4**_**Se or only Na**_**2**_**O**_**4**_**W and a 6-day culture with no Na**_**2**_**O**_**4**_**W + Na**_**2**_**O**_**4**_**Se or only with Na**_**2**_**O**_**4**_**Se (Mean and standard deviation were calculated for 10 independent culture tests for each condition)**

	**48-hour culture**			**6-day culture**	
	**without**	**with**	**with**	**with**	**without**
	Na_2_O_4_W + Na_2_O_4_Se	Na_2_O_4_W + Na_2_O_4_Se	Na_2_O_4_W	Na_2_O_4_Se	Na_2_O_4_W + Na_2_O_4_Se
Means	2.13E + 10	4.42E + 12	3.93E + 12	4.02E + 12	3.22E + 12
Standard deviation	5.56E + 09	1.84E + 11	3.67E + 11	2.23E + 11	1.53E + 11

**Figure 1:**
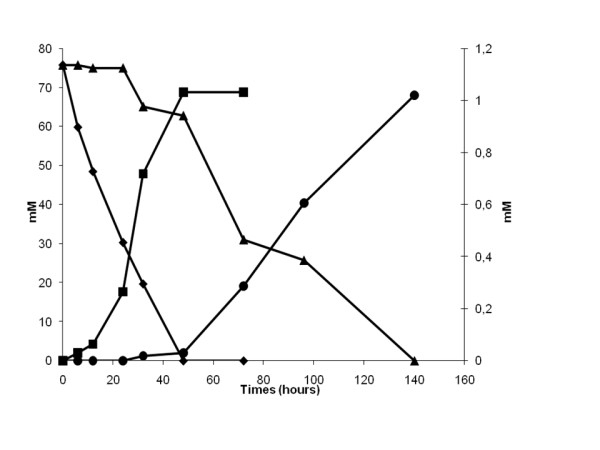
**Visualizations of H**_**2**_**used (in mM; left Y axis) and CH**_**4**_**(in mM; right Y axis) produced by *M. stadtmanae* with and without addition of sodium tungstate solution (Na**_**2**_**O**_**4**_**W) (over 140 hours (X axis).** ♦ H_2_ used with sodium tungstate (Na_2_O_4_W), ■ CH_4_ production with sodium tungstate (Na_2_O_4_W), ▴ H_2_ used without sodium tungstate (Na_2_O_4_W), and ● CH_4_ production without sodium tungstate (Na_2_O_4_W).

**Figure 2:**
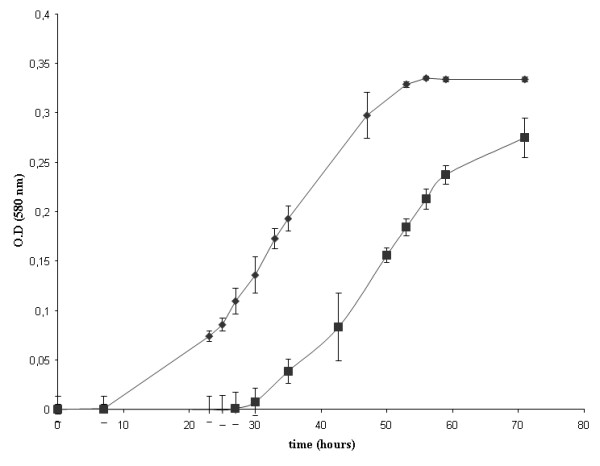
**The effect of addition of selenite/tungstate solution on growth of *M. stadtmanae.* ** ♦ Growth of *M. stadtmanae* with tungstate (Na_2_O_4_W). ■ Growth of *M. stadtmanae* without tungstate (Na_2_O_4_W).

The addition of sodium selenate alone has no effect on the growth curve of *M. stadtmanae*. However, the addition of sodium tungstate alone or in combination with sodium selenate shortened the lag period to 2 days post-inoculation with an equivalent 16S rRNA and *rpo*B genes copy number and with equivalent rates of methane production (Figure 1). In the absence of tungstate, *M. stadtmanae* exhibited a 30-hour log phase. Adding tungsate to the culture medium reduced the delay of this log-phase so that it took 47 hours instead of 72 hours to achieve a 0.35 optical density of the culture (Figure 2). These results correlated with the fact that *M. stadtmanae* genome encodes a formylmethanofuran dehydrogenase comprising of five sub-units (Genes IDs: 3855499-3855500-3855501-3855502-3855503), an enzyme found in methanogenic *Archaea*. In strict anaerobic micro-organisms, this enzyme catalyzes the reversible dehydrogenation of formylmethanofuran into CO_2_ and methanofuran. The formylmethanofuran dehydrogenases are either molybdenum- or tungsten-iron-sulfur proteins. The tungsten is likely bound to the same skeleton as the molybdenum in the so-called molybdopterin dinucleotide cofactor [8-10].

Previous reports described the requirement of tungsten for growing numerous methanogens including *Methanothermobacter wolfei* which has an obligate requirement for tungsten to maintain autotrophic growth, *Methanococcus vannielii* requiring tungsten as a cofactor for the enzyme formate dehydrogenase [11], *Methanogenium tatii*[12] and *Methanocorpusculum parvum*[13] also requiring tungsten for growth (Table 2). Selenium has also been reported as stimulatory and may be required for many methanogens, especially members of the genus *Methanococcus* as *Methanococcus vannielii*[11], *Methanococcus jannaschii*[14], *Methanococcus maripaludis*[15], *Methanococcus voltae*[16] and *Methanococcus thermolithotrophicus*[17] (Table 1). Requirement for selenium could have enzymatic basis, since it was reported that *M. vannielii* possesses a selenium-dependant formate dehydrogenase [18]. Selenium was also reported as a component of both a hydrogenase [19] and tRNA [20].

**Table 2:** Requirement of tungsten or/and selenium for growth of methanogens as reported in bibliography

**Species**	**Tungsten**	**Selenium**	**References**
*Methanothermobacter wolfei*	**YES**	**NA**	[21]
*Methanococcus vannielii*	**YES**	**YES**	[11,18]
*Methanogenium tatii*	**YES**	**NA**	[12]
*Methanocorpusculum parvum*	**YES**	**NA**	[13]
*Methanococcus jannaschii*	**NA**	**YES**	[14]
*Methanococcus maripaludis*	**NA**	**YES**	[15]
*Methanococcus voltae*	**NA**	**YES**	[16]
*Methanococcus thermolithotrophicus*	**NA**	**YES**	[17]

In the absence of tungstate, *M. stadtmanae* exhibited a growth delay of 5–7 days which is long for testing *in vitro* susceptibility to antibiotics [22]. As we now observed that tungsten enhances the growth of two taxonomically unrelated methanogens, *M. stadtmanae* and *M. luminyensis*, we suggest that tungsten-containing media could be incorporated into the panel of media used for the isolation and culture of new methanogens from clinical and environmental specimens, and for testing their *in-vitro* susceptibility to antibiotics.

Methanogenic *Archaea* recently emerged as normal components of the human gastrointestinal and oral microbial ecosystems, where they could play important roles in health and diseases [23]. However, the isolation of such organisms requires long incubation times and strict anoxic atmosphere and is hampered by the incomplete knowledge of their nutritional requirements [23]. In fact, the result obtained in the present study may prompt further phenotypic characterization including extended antibiotic susceptibility testing [22] and even allowing isolation of new *Archaea* in order to assess understanding their contribution in the physiology of complex human microbiomes and their potential role in the course of infections.

## Competing interests

The authors declare that they have no competing interests.

## Author’s contributions

BD, SK, MLF designed and performed analyses, BO, MD interpreted data and wrote the draft. All authors read and approved the final manuscript.
